# Synthesis, characterization, and antimicrobial evaluation of polyvinylalcohol-osthol composite films

**DOI:** 10.55730/1300-0527.3496

**Published:** 2022-08-30

**Authors:** Ishrat GOWSIA, Feroz A. MIR, Javid A. BANDAY

**Affiliations:** 1 Department of Chemistry, Natural Product Research Lab, National Institute of Technology Srinagar, Srinagar, India; 2 Department of Physics, Baba Ghulam Shah Badshah University Rajouri, Rajouri, India

**Keywords:** Natural products, osthol, PVA polymer, food packaging, antimicrobial activity

## Abstract

Although poly-vinyl alcohol (PVA) has certain mechanical drawbacks such as a weak barrier, it has widely been used in food packaging over the last many years. To increase the suitability of PVA (C_2_H_4_O)_n_ and render it ideal for food packaging, a diversity of studies have already been carried out. In the below-mentioned script, we, for the first time, report the use of natural product osthol in making a new composite with PVA for enhancing thermal, physicochemical, and antimicrobial properties. The significant aim of the report is the insertion of osthol (C_15_H_16_O_3_) into PVA polymer, which is to be subsequently used for antimicrobial applications. The synthesis of the polymer composite film is done by solvent casting method and is characterized by SEM, XRD, FT-IR, and UV-Vis spectroscopy analysis. The manifestation of antimicrobial activity against (*S. aureus*) (ATCC8738P), *E. coli* (ATCC8739), *Aspergillus niger*, *Alternaria alternata*, and *Fusarium solani* by the film composite is remarkable. The addition of osthol molecule increases the tensile strength of PVA films from 18.73 ± 0.56 Mpa (PVA) to 24.58 ± 0.49 Mpa (15 mL). As a result, tensile strength increases by 23.79% in a film containing a higher concentration of osthol (15 mL). The barrier properties of PVA osthol composite films improve with the incorporation of osthol. OTR and WVTR decrease by 43.03% and 30.24%, respectively, on the addition of 15 mL osthol. Reduction in OTR and WVTR of the films could increase their applicability in the food sector. An increase in contact angle from 43° (pure PVA) to 66.7° increases the hydrophobic character of the composite films which is desirable for food packaging. This noticeable enhancement of the properties of the PVA film like hydrophobicity, mechanical, barrier, and antimicrobial is supporting the potential application of achieved material in packaging of easily perishable foods like fruits and vegetables by extending their shelf life.

## 1. Introduction

Petro-based synthetic polymers have globally alternative applications such as packaging, appliances, building, and construction. And these applications are considerable. About 42% of polymers in the global polymer market are used for packaging food, chemicals, cosmetics, and pharmaceuticals and this ratio increases by 5% annually [1–3]. Owing to their blatant features, polymeric materials have been used in recent times as a worthy substitute for metals, glass, and ceramics [4–6]. The splendid mechanical and barrier properties, feasible processability, economic viability, and prevalent availability of synthetic polymers such as polystyrene (PS), polyethylene terephthalate (PET), polyamide (PA), polyvinylchloride (PVC), polypropylene (PP), and polyethylene (PE) have eminently framed a motive for their widespread use in packaging applications since the middle of the 20th century. Meanwhile, the poor biodegradability of synthetic polymers has always been an anxious hindrance to their futuristic prevalence [1–2,4]. Noteworthy research work has been motivated by increasing concerns about the environmental impact of nondegradable polymers with the visionary objective of finding a solution to reduce plastic waste. Adequate approaches have been used to eliminate this environmental issue which encompasses landfills to store the waste, burning, and recycling of plastic waste. Worsening growth of urbanization, global warming, high costs, and energy consumption inevitably pave hindrances for any further reduction of plastic wastes in an eco-friendly manner. The researchers, therefore, have come forward with the idea of replacing the nondegradable polymers with fully biodegradable alternatives in a more realistic manner [7–12]. There has been a significant increase in the number of publications associated with this research area since 2000. As per the global market data for polymers, biodegradable polymers pooled for approximately 1% of total consumed plastics in 2009, but this may reach 20% by 2020 [13]. Unfortunately, the pathetic material performance of most biopolymers, particularly with regards to their barrier properties, dear prices, and material processing problems, has so far hindered their application as neat packaging materials. Instead of this, biopolymers are usually combined with other synthetic polymers or else reinforced with different fillers intending to overcome this limitation [4,14–16]. Being an artificial, semicrystalline, water-soluble polymer, PVA is distinguished for having a broad spectrum of applications [17]. Many studies, therefore, have been concerned with the material engineering of PVA for the betterment of its credentials. The properties are greatly enhanced with the incorporation of nanosized reinforcement, nanofillers such as titanium dioxide, zinc oxide, silicate, clay, silver nanoparticles, and antimicrobial agents of natural extracts.

PVA also owes its popularity to the status of being one of the most popular synthetic biopolymers for packaging applications and is the cardinal subject of this paper due to its good biodegradability, considerable reluctance to oxygen, biocompatibility, film-forming capacity, good transparency, easy processability, and desirable mechanical and thermal properties. PVA has also been approved for use in packaging meat and poultry products by the USDA [18]. Pure PVA has a more hydrophilic character and low biodegradable character, which limits its use in packaging as pure polymer; hence, scientists have made its blends and composites to overcome such limitations. Blends of PVA and starch have resulted in improved biodegradability [19–25]. Similarly, composites of pure PVA and organic acids have decreased the hydrophilicity of polymer [26]. Composite materials of PVA and natural products have resulted in additional properties like antimicrobial and antioxidant properties [27]. Nanocomposites of PVA containing nanofillers of inorganic substances like Ag have resulted in enhancement of a variety of properties like thermal and antimicrobial properties. Cagatay Altinkok et al. fabricated nanocomposites of Ag nanowires and poly(1,4 cyclohexanedimethylene acetylene dicarboxylate via aza Michael addition reaction for antimicrobial applications [28]. Mohanty et al. reported that biocomposites of PVA-date palm leaf fibre (DPL) fabricated via melt mixing route occasioned in the enhancement of properties like mechanical, compatibility, and thermal properties. An increase in young’s modulus of the biocomposite from 362.5 to 1183 MPa was observed with 28 wt% amounts of DPL [29]. Similarly, Singha et al. observed improvement in mechanical, thermal, and antimicrobial properties of corn starch-PVA biocomposite containing lignin-free Grewia Optiva fibres. Based on their antimicrobial properties, they could be used in the food industry for packaging applications [29]. In other studies by Tran et al., augmentation in the properties like thermal, mechanical, and antimicrobial of microalgae ash-PVA biocomposites was reported [30]. Furthermore, the studies suggest their use as antimicrobial ones.

There are several methods employed for small and large-scale production of PVA composite films and blends like solution casting method, melt processing, extrusion, and injection molding. However, the solution casting method is the most common method due to less thermal degradation of polymers and organic compounds at low temperature and shear [31].

Taking the above facts into account, this work comprises synthesis of PVA ostholcomposite film by solvent casting method. The osthol molecule{7-Methoxy-8-(3-methylbut-2-enyl)chromen-2-one} or 7-methoxy-8-(3-methylbut-2-enyl)-2*H*-1-benzopyran-2-one), one of the major constituents of various *Prangos* species in general, and *Prangos pabularia* in particular, is a simple coumarin. This molecule is biologically active and has good antibacterial [32–33] and antifungal activity [34]. The film composite is fully characterized and its thermal and antimicrobial properties are evaluated. Thus, it has the potential to act as a natural food preservative in enhancing the shelf life. This report on the use of osthol for the synthesis of a film composite with PVA has been formed for the first time.

## 2. Materials and methods

PVA was procured by HiMedia (India). According to the procedure mentioned in our previous work, extraction of Osthol from the *Prangos pabularia* plant was made [35].

For the preparation of 5% (w/v) solution of PVA, 5 g of PVA was dissolved in 100 mL of distilled water. This solution was heated at 75 °C on a magnetic stirrer for 3 h to get a transparent solution [36]. The solution was placed inside the sonicator for 15 min to get rid of air bubbles if present. Next, 25 mL of this solution was cast on a Petri plate for drying. This dried film was then peeled off and placed inside the desiccator for further characterization.

For the preparation of PVA osthol composite films (PO), the preparation of two solutions (**Solution A & Solution B**) to be subsequently used for PO film composite was a part of our proceedings [37]. **Solution A** 0.045 wt% was prepared by the dissolution of 15 mg of osthol in 30 mL DMSO. **Solution B** 5 wt% was prepared by dissolving 5 g of PVA in 100 mL distilled water, heated perpetually at 75 °C for 3 h at a stretch, and homogeneity and clarity were thereof made sure. Consequently, tantamount volumes of **solution B** were added to 5 mL, 10 mL, and 15 mL volumes of osthol **(OL)** solution. The weight percentages of the above three solutions are 0.0076%, 0.013%, and 0.017%. After that, each solution was heated at 80 °C on a magnetic stirrer for 2 straight hours. Eventually, the three solutions were transferred to Petri plates for parching, which took almost 3 to 4 days to get implicitly dry composite films. Lastly, the prepared films were peeled off from Petri plates and packed in black paper sheets to evade solar exposure. Finally, these films were kept inside a desiccator for further characterization.

**Figure 1:**
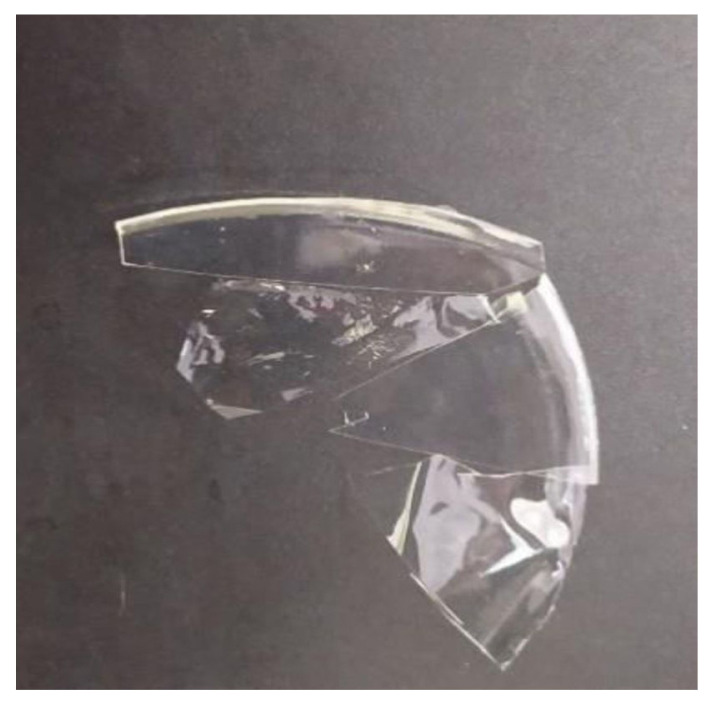
Pure PVA film.

**Figure 2:**
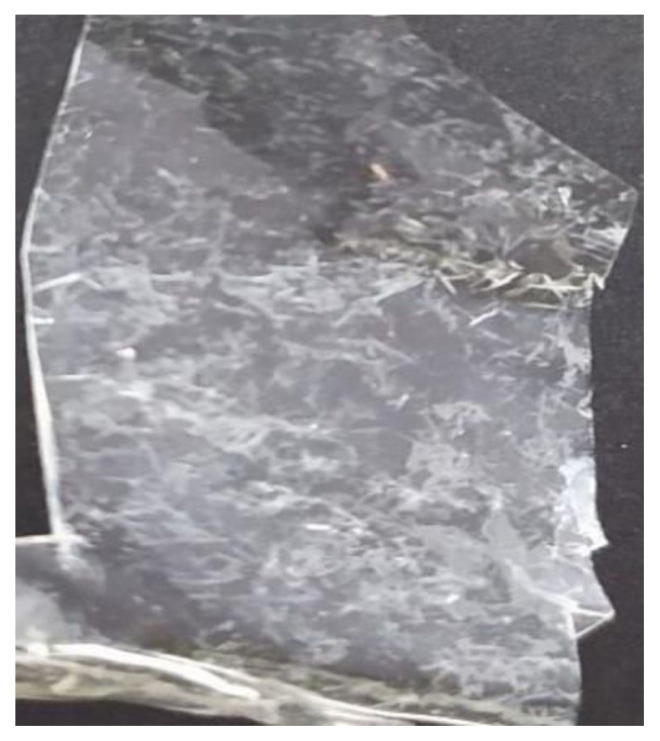
Osthol-incorporated PVA film (15 mL).

Pure PVA film was found to be highly smooth and transparent. With the incorporation of osthol, the film became translucent with a rough surface and a slight increase in thickness. Visually needle-like structures were distinguished. Needle-like structures confirm the deposition of osthol on the polymer surface as is shown in Figures 1 and 2.

Perkin Spectrum 2 was used for FTIR studies of prepared polymer composites. The XRD was carried out using (Rigaku smart lab 9 kW rotating anode X-ray diffractometer; Cu Kα radiation of wavelength (λ) = 1.54050 Å) for crystal structural studies of the sample. The field emission scanning electron microscopy (FESEM) ZEISS Gemini SEM 500 operated at 15 kV was used for surface morphological studies of polymer composites.

Shimadzu UV-1601 spectrophotometer was employed for optical properties in the wavelength range of 200 nm to 800 nm. Film absorbance at 600 nm was used to measure the opacity of films as per the method given in [38]. A spectrophotometer was used to place rectangular film. This was done in reference to a blank test tube. The opacity of the films was calculated by the following equation:


(1) $$T=\frac{A_{Abs.600}}{\chi },$$

where T stands for transparency, Abs 600 is the absorbance at 600 nm, and χ is the thickness (mm) of the film. According to the above equation, the high values of T indicate lower transparency and a higher degree of opacity.

Mettler Toledo DSC/TGA instrument was implemented to carry out the study of thermal properties. Under the influence of flowing nitrogen, the heating rate was kept at 10 °C/min to perform TGA measurements.

Microscope MicroView (USB Digital Microscope) supported with an image analysis software was employed in the determination of the wettability property of the surface of the films using contact angle ϴ. The film surface was treated with distilled water of 2 μL quantity. The measurement of the contact angle formed by the intersection of the liquid-solid interface (a drop of water-surface of the film) and the liquid-vapor interface (tangent on the boundary of the drop) was carefully calculated [39]. The final measurement was taken as the mean of five different measurements.

Implementation of the Drying Oven Method was made to obtain the moisture content. Initially, 2 × 2 cm^2^ film pieces were cut and weighed (*W_1_*). The film pieces were dried at 105 °C in a hot air oven for 24 h. Finally, dry weight (*W_2_*) was obtained [40]. The moisture content (MC) was calculated from the weight loss using Eq. (2):


(2) $$MC=\frac{W1-W2}{w1}\times 100.$$

For determination of the solubility of the film (FS), the methodology given in [40] was performed. Before the calculation of the percentage of dissolved dry matter, films were immersed in water to accurate the experiment. Film samples of dimensions 2 cm × 2 cm were cut out. These samples were later on dried at 60 °C for 24 h. This was done to determine the initial dry weight (*M_i_*). Film samples were dipped in deionized water in 30 mL quantity. This was followed by mild shaking for 24 h. The samples were then removed and dried at 100 °C for 24 h to determine the undissolved final dry weight (*M_f_*). The % film solubility (FS) was calculated using Eq. (3):


(3) $$\text{FS}=\left|\frac{M_{i}-M_{f}}{M_{i}}\right|\times 100.$$

The standard method of ASTM E 96-95 with modification was exercised in the determination of the water vapor transmission rate (WVTR) [41]. Eighteen millilitres of distilled water was filled in a WVP measuring cup. For prevention of water vapour loss, film samples of dimensions (7.5 cm × 7.5 cm) were used to cover the cup and clamp it strongly. The assembled WVP cup was weighed and subsequently placed in a controlled environmental chamber set at 25 °C and 50% RH. Weight change of the cup was observed periodically after every 1 h for 8 consecutive hours. For calculation of the water vapour transmission rate (WVTR; g/m^2^.s) of the film, the slope of the steady-state (linear) portion of the weight loss versus the time plot was used.

The oxygen transmission rate (OTR) measuring system (Labthink; C230) according to ASTM D3985—17 standard at 23 °C and relative humidity of 0% was implemented in the determination of oxygen transmission rate. Two parallel samples were tested for each packaging film.

Calibrated digital micrometre Vernier calliper was used for thickness measurements at three different places of the film. Next, an average of the three measurements was taken. Standard mechanical properties of composite films such as tensile strength, and elongation at break %, as per ASTM D, 882, were measured by making use of Tinius Olsen H50 KT universal testing machine. This was done by using a 5 kg (50N) load cell at the crosshead speed of 5 mm/min. Film samples of dimensions 5 × 1 cm^2^ were accommodated between two gripping units of tensometer, with a 3 cm gauge length left for mechanical loading. From the results of five tests, average values are observed and expressed as mean ± standard deviation (SD).

An evaluation of the antibacterial activity of PVA osthol composite against two bacterial strains *E. coli* and *S. aureus* was done by using the liquid test culture method [42]. In the liquid test culture method, circular composite films of 20 mm diameter were cut, sterilized by UV radiations for 20 min, and immersed in tryptic soy broth (TSB) medium in four tubes. Next, 100 μL of bacterial inoculum (~10^8^ CFU/mL) was added to the TSB medium in four tubes in which films were already placed. This was kept in a shaker incubator (200 rpm) at 37 °C for 24 h. After that, 1 mL of the sample was withdrawn and ten-fold diluted with maximum recovery diluent media. These ten-fold dilutions were spread on trypticase soy agar (TSA) medium by spread plate method. The plates were kept in an incubator at 37 °C for 24 h to get the colony-forming unit (CFU/mL) to count.

Composite films of osthol were evaluated for their antifungal activity against mycelial growth of pathogenic fungi *Aspergillus niger*, *Alternaria alternata*, and *Fusarium solani* by food poisoning technique [34]. The 20-mm circular films, first sterilized under a UV chamber for 15 min, were mixed with autoclave sterilized potato dextrose agar. Subsequently, 15-mL mixtures were transferred into sterile Petri plates and allowed to solidify. This was all done under a laminar airflow chamber to ensure aseptic conditions. After solidification, these Petri plates were inoculated by placing 2-mm mycelial discs of the above fungus in the centre of each plate. Actively growing colonies were used for the purpose. Three replicates were maintained for each film. Petri plates were incubated at 24 ± 2 °C. Colony diameter was measured and recorded after 3 days. PDA plate without polymer osthol composite is served as a negative control. The percentage inhibition of fungal growth by these composite films can be obtained by Vincent formulae:


(4) $$I=\frac{C-T}{C}\times 100,$$

where I is the percent inhibition, C is the colony diameter in control, and T is the colony diameter in treatment.

## 3. Results and discussion

### 3.1. UV-Vis. absorption analysis

**Figure 3:**
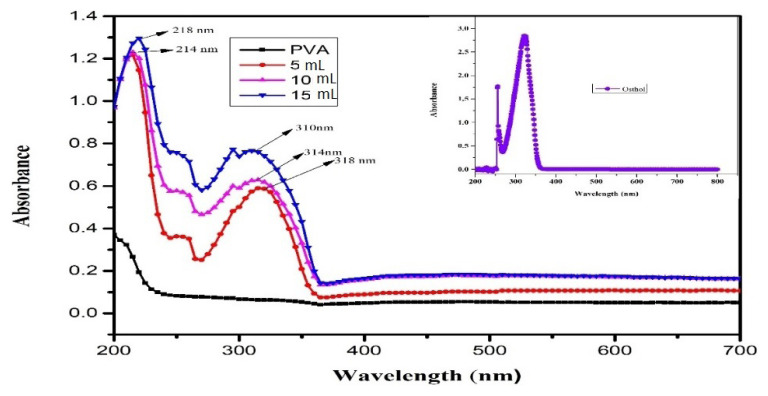
UV-Vis absorbance spectrum of pure PVA and PO film composite at different concentrations of osthol.

The absorption spectra of **PVA**, osthol, and **PO** film are given in Figure 3. The spectrum of pure **PVA** did not show any distinguishing peak in the UV visible region of the spectrum. The UV spectrum of an osthol molecule is showing absorption peaks at 322 nm and 254 nm in inset Figure 3. However, after doping **PVA** with osthol (**OL**), the composite film (**PO**) displayed distinctive absorption peaks from 218 nm to 318 nm, which confirmed the incorporation of **OL** in **PVA**. The absorption bands intensified and shifted towards the left as the concentration of osthol increased [43]. High polyphenolic compounds containing natural extracts and essential oils exhibit the same observation [44]. The interaction between the two is well confirmed by the shifting of the peaks towards a lower wavenumber.

The opacity of the films was measured as per equation (1) at 600 nm wavelength. PVA films without osthol were found to be more transparent or to have lower opacity values than those containing osthol (Table 1). The reason for the increase in opacity of composite films could be due to the light absorption ability of osthol molecule. Since the osthol molecule contains a benzene ring fused with a pyran ring (benzopyran moiety with a carbonyl group at carbon number 2). The presence of the benzene ring, the carbonyl group favours n-π* absorption in the UV region. As a consequence, these films have excellent UV barrier properties. Hence, these films could act as a protective and preventive fence to UV light and could prevent photodegradation of fat-rich foods [27]. Hu et al. detailed enhancement in the UV light barrier of gelatine films by impregnation of *Ginkgo biloba* extract, while Vilela et al. reported improvement in the light barrier property of chitosan films by ellagic acid [45,46]. A significant increase in film opacity was obtained with an increased concentration of osthol [47].

### 3.2. FT-IR analysis

**Figure 4:**
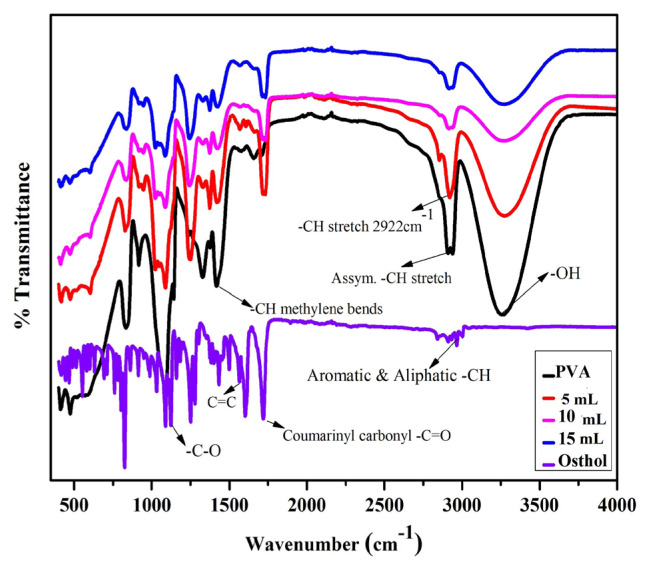
FT-IR analysis of osthol (OL), PVA, and PO composite film with different concentrations of osthol.

The spectra of PVA, osthol, and their composite films are illustrated in Figure 4. The FT-spectrum of pure osthol shows a typical peak at 1723 cm^−1^ which is the characteristic peak of coumarinic carbonyl. Aliphatic & aromatic C-H bonds are observed in the bands between 2700 and 3000 cm^−1^. Aromatic C=C exhibits the peaks at 1490–1600 cm^−1^. Another absorption peak around 1130 cm^−1^ corresponds to the C-O stretch. Owing to the characteristic stretching vibrations of O-H groups involved in inter- and intramolecular H-bonding between polymer chains, the pure PVA spectrum showed a strong and broadband at 3263 cm^−1^. Due to electrostatic weak interactions between PVA −OH electric dipoles and aromatic C-H stretches of osthol this band commence broadening with the incorporation of osthol in the polymer matrix [37,43]. A shift in the band corresponding to asymmetric −C-H stretches from 2926 cm^−1^ to 2922 cm^−1^is observed. The widening and shifting of the peaks between these two molecules are influenced by hydrogen bonding [37].

The band at 1428 cm^−1^ corresponds to C-H bends of methylene group vibrations of PVA [48]. The consequently decreased intensity at this band in the composites by decoupling supports the interaction between the two [36]. Again, peak merging at 1710 cm^−1^ (-C=O) of pure PVA and 1723 cm^−1^ of osthol shows hydrogen bonding type of interactions between the two [37]. Concisely, the changes in PVA structure with osthol incorporation show consistency with the given XRD data.

### 3.3. XRD analysis

**Figure 5:**
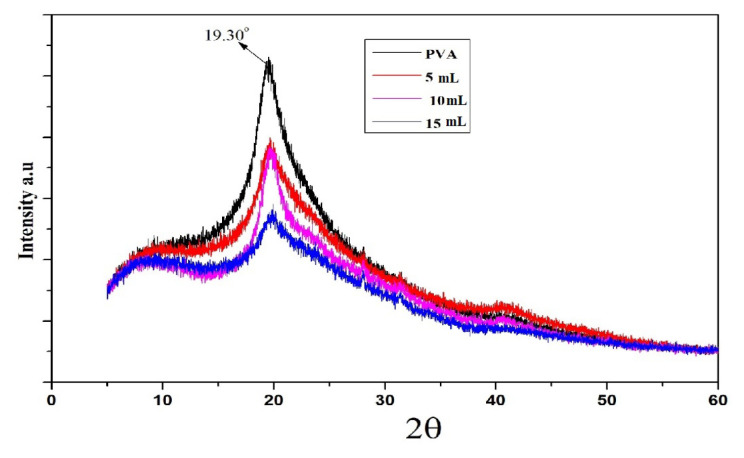
XRD analysis of pure PVA and PO composite film at different concentrations of Osthol.

The XRD analysis of virgin **PVA** and **PO** composite film is shown in Figure 5. In XRD analysis, the PVA film displayed a major diffraction peak at 2θ = 19.68° [49]. This peak observed in PVA corresponds to the (101) crystal plane of polyvinyl alcohol and inferred its semicrystalline nature [50]. It can be seen from the graph that individual peaks of the osthol molecule are missing [51]. However, as the volume of **OL** augments, the intensity of the characteristic peak of PVA decreases, thereby indicating the loss of the semicrystalline nature of PVA into amorphous. Besides, the characteristic peak broadens due to polymer complex formation. This, in turn, results in the polymer chain separation and different rearrangements in their structure. Also in our dopant molecule, we have hydrogen and oxygen atoms, which can interact via van der Waals and weak interactions with polymer hydrogen atoms [37]. The interaction causes a significant decrease in crystallinity in the host molecule. Another reason for decreased crystallinity may be due to the heat-induced alterations (>70 °C) during the preparation of the composite [49]. In addition, the lattice points like phonons are moved by the supplied heat. Due to this movement, existing bonds break off and lattice systems give rise to new bonds [49]. FT-IR data, as mentioned before, provides an evidential aid to the interaction between OL and PVA and infers the noncovalent nature of this interaction.

### 3.4. Field emission scanning electron microscopy

**Figure 6:**
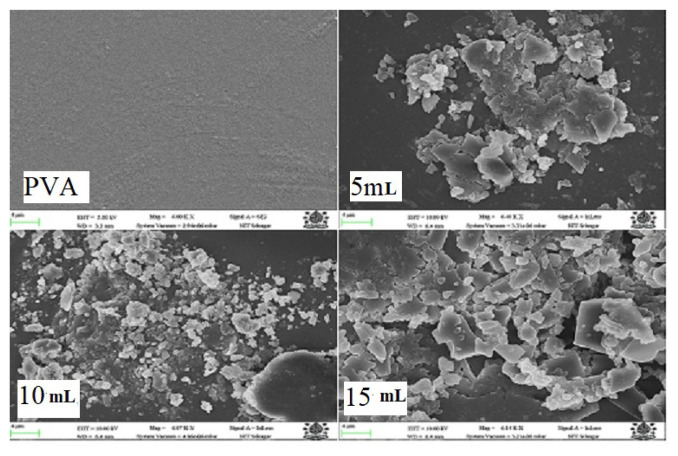
SEM analysis of PVA and PO composites with different concentrations of osthol.

FESEM gives us micrographs of pure PVA and its composites with different concentrations of natural product osthol molecule in Figure 6. These exhibit morphological changes on the surface of pure PVA polymer after doping with the osthol molecule. From these micrographs, it is clear that PVA is clear and free from white particle deposition. Because of the presence of an osthol molecule, white particles are found dispersed on the surface of the polymer matrix leaving its surface rough. The surface properties like hydrophobicity are proportional to the degree of roughness of the surface. Surfaces with more roughness, exhibit more hydrophobic character and such surfaces, thereof, might be employed in food packaging [52]. These white particles are found to increase with an increased amount of osthol on the polymer matrix. A similar observation was well supported by several studies [50,53]

### 3.5. Thermo-gravimetric analysis

**Figure 7:**
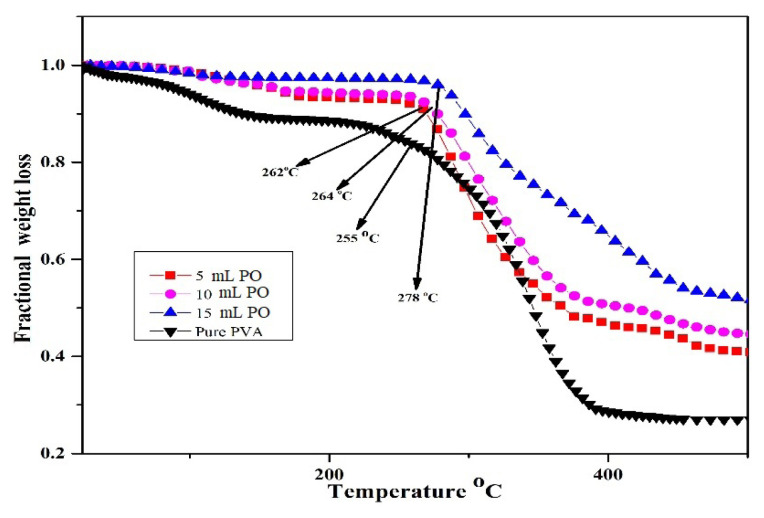
Fractional weight loss with temperature.

As the polymeric materials to be employed in food packaging are required to survive high heat and moisture retort treatments, they are necessitated to have considerable thermal stability. Thermal and oxidative stability, chemical structure, chemical composition, and water activity of films are determined by the TGA test. Examining the alteration in thermal degradation peak, therefore, turns out to be an advantageous technique in verifying the incorporation of bioactive components in polymer films. Hydrogen bonding between −OH groups in pure PVA polymer is responsible for interchain and intrachain interactions between polymer chains. These existing interactions between polymer chains in pure polymer could get changed due to the presence of the methoxy group present in osthol PVA composite and thus could modify the physical structure and crystalline behaviour. The TGA curves of pure PVA and osthol-PVA composites are shown in Figure 7. In these curves, weight loss occurs in three steps. Initially, weight loss is due to the evaporation of water molecules; thus, moisture content can be obtained from the initial weight loss curve [54]. The weight loss in the second stage can be due to decomposition, thereby giving decomposition temperature of PVA which can be due to breakage of basic vinyl alcohol units and bond cleavage in the PVA backbone. In the third step, there occurs the fragmentation of the giant structures of both PVA and osthol [54,55]. The TGA curves show that the thermal stability of osthol embedded PVA films has improved as compared to neat PVA. The improvement in the thermal stability could be due to interactions between OH groups of PVA and the OCH_3_ group of osthol [56]. The reason for improved thermal stability can be explained by the reduced movement of PVA chains which manifested in the repression of chain transfer reaction and continuously slowing down degradation processes [57]. Pour et al. found increased thermal stability of starch PVA composite film containing citric acid and suggested cross-linking interaction of critic acid molecules with starch and PVA chains [58]. The thermal stability of polymer films is augmented with the inculcation of osthol in it. This augmentation was appreciable from the fact that the degradation temperature altered from 255 °C to 278 °C. The augmentation in thermal stability must be quite beneficial in the production of films via extrusion/compression molding. It may henceforth be inferred that the natural products assist in the enhancement of utilization of edible films for food applications. Furthermore, it can be concluded that natural products enhance the functionality of edible films for food applications. The exhibition of the higher crosslinking, density, and lower porosity of films with good oxidative stability is very well appreciable with the increment in thermal stability. The moisture content obtained was 10.18%, 6.20%, 5.37%, and 1.86%, respectively, for pure polymer 5 mL, 10 mL, and 15 mL composites of osthol from the first weight loss step.

### 3.6. Contact angle

**Figure 8:**
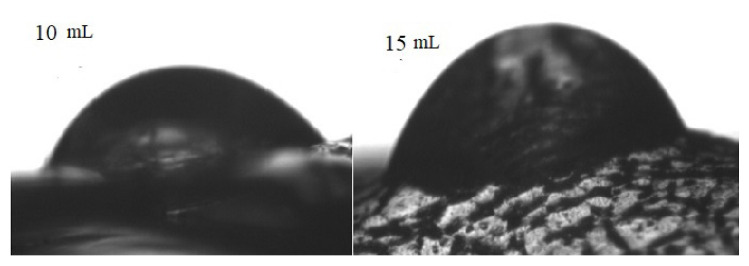
Image of a water droplet on the film surfaces of (a) 10 mL (c) 15 mL.

Uneven surfaces are found to have a more hydrophobic character than smooth surfaces [50]. Similarly, contact angle, hydrophobicity, and surface roughness are in direct relationship with one another, implying that rough surfaces have more contact angle and increased hydrophobic character [59, 60]. The contact angle increases with the irregular surface as roughness increases the part of air captured within the surface [61, 60]. The contact angle of the PVA osthol films is given in Figure 8. From the SEM micrographs of polymer composites, it is clear that increasing osthol concentration is increasing the roughness of the films, hence the contact angle. The terms “hydrophobic” and “hydrophilic” are respectively defined as θ > 65° and θ < 65° [62]. The contact angle for pure PVA is 43°. The contact angles for 10-mL and 15-mL films increase from 43° to 65.2° and 66.7°, respectively. In the case of 5-mL composite film, the increase in contact angle was not significant. Hence, the PVA film after being incorporated with 10 mL and 15 mL of osthol has developed a hydrophobic nature. The hydrophobic nature of PVA osthol can crucially be benefited in food packaging. Unlike the behaviour observed in roughness tests, the increase in the concentration of osthol did not affect the value of the contact angle considerably. Therefore, the relationship between contact angle and surface roughness can be acquired by doing a comparison between 15-mL composite film with the polymer matrix and not between 15-mL and 10-mL composite films [63].

### 3.7. Moisture content

The films with hydrophobic surfaces (10-mL and 15-mL composite films) as supported by contact angle measurements exhibited lower values of the moisture content as in Table 1 compared to pure PVA. For 15-mL PO composite film, a reduction of MC was observed when compared with the neat PVA. This could probably be due to noncovalent interactions between the polymer network and the osthol molecule which could have controlled the access of hydroxyl groups for hydrogen bonding with water molecules [38]. As a consequence of which, the affinity of the films towards water has decreased.

### 3.8. Film solubility

Insolubility of film in water is a prerequisite for ensuring the retention of the seminal qualities of food products [64]. The solubility of the film is an indicator of the water resistance of the film. Since pure PVA film is entirely soluble in distilled water, a need arises to overcome this solubility. The addition of variable concentrations of osthol in PVA films does the task of reducing the solubility of films. To exemplify this, we take note that the solubility of 15-mL PVA-osthol films was found to be lower than that of pure PVA film. The results in Table 1 show that the water resistance is more in those PVA films which have a higher concentration of osthol. This inference may well be attributed to the hydrophobic nature of the film. It was found that in the case of PVA film containing starch, glycerol, and halloysite nanotube increase in contact angle shows a correlation with a significant reduction in water solubility of nanocomposite film with increasing wt% of halloysite nanotubes [31].

### 3.9. Water vapour transmission rate and oxygen transmission rate

To cater to the maintenance of the quality of the food products, materials recruited in packaging need to have seminal oxygen and water barrier properties. Materials with both seminal oxygen and water barrier properties do not, however, show their presence in nature. Generally, olefin polymers possess immaculate moisture barrier properties. This owes to the fact that these polymers contain nonpolar functional groups in the repeating units, e.g. polyethylene and polypropylene. PVA has good oxygen barrier features due to its crystalline nature and intermolecular attraction between hydroxyl groups in monomeric units. The barrier properties of PVA osthol films were observed as a function of increasing volumes of an osthol molecule or wt% of osthol as shown in Table 2. The oxygen transmission rate (OTR) value for pure PVA is 9.04 cc/m^2^/day. The OTR value for PO films varies from 7.78 to 5.15 cc/m^2^/day. This indicates OTR of virgin PVA films was reduced by the incorporation of osthol. A similar result of a reduction in OTR values has been observed with PVA-based PVA boric acid hybrid films [65].

Chemical structure, film morphology, nature of the permeate, and measurement conditions such as temperature and water vapour pressure gradient are the credentials that govern water vapour permeability [66]. The water vapour transmission rate of packaging materials should, normally, not exceed 1000 g/m^2^/day. The aforementioned water vapour transmission rate shields food against the accumulation of excessive moisture and consequently against the redundant growth of microbes associated with that excessive moisture. The WVTR of pure PVA film, as mentioned in Table 2, is 958.66 g/m^2^/day. With an increment in the concentration of osthol, this value keeps on altering in descending order from 958.66 to 668.67 g/m^2^/day. The lower value of WVTR of 15-mL composite film compared to pure PVA might be attributed to the hydrophobic nature of the film. Hydrogen bonding interactions between hydroxyl groups of PVA and osthol lower OTR and WVTR of the PVA-osthol films. Since the availability of free hydroxyl groups of polymer for hydrogen bonding with water decreases with this interaction, it could, therefore, enhance its barrier properties. By incorporation of green tea extract into chitosan-based films, a similar inference was obtained by Siripatrawan [38]. The reduction of water vapour permeability in green tea extract is a result of hydrogen and covalent type interactions between chitosan and polyphenolic compounds of it. Similarly, Gómezguillin et al. observed a reduction in the number of hydroxyl groups available for hydrogen bonding with water due to cross-linking reaction between antioxidant extracts from murta leaves and tuna-fish gelatine [67].

### 3.10. Thickness and mechanical properties

The thickness of the composite films, as depicted in Table 1, varies from 0.126 to 0.156 mm. Pure PVA is found to be the thinnest.

**Figure 9:**
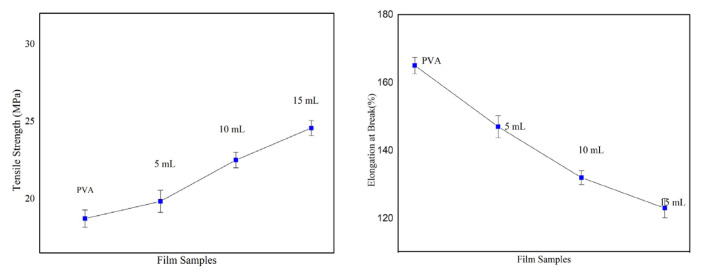
Tensile strength and % elongation at break of film samples.

Mechanical properties such as tensile strength (TS) and elongation at break (EB) play a pivotal role in regulating the quality of food packaging materials. A universal testing machine was employed to infer the mechanical properties of different PO films. As illustrated in Figure 9, the mechanical properties of the PVA film ameliorated with the inclusion of osthol into pure PVA film. Given in Table 2, tensile strength of virgin PVA is enhanced with increasing wt% of osthol into PVA, whereas the % elongation at break was reduced. While the tensile strength of pure PVA is 18.73 ± 0.56 Mpa, the PO composite films exhibited an increasing trend from 19.84 ± 0.72 Mpa to 24.58 ± 0.49 Mpa. Reduction in EB is preferable in food packaging, as this property is directly related to the biodegradability of the films [68]. As the osthol content increases, elongation at break decreases from 165 ± 2.45 to 123 ± 2.86; thus, a decreasing trend is observed in the % elongation of PO hybrid films. As food packaging materials have to approach common and mediocre stress during handling, food storage, delivery, and transportation, improved tensile strength is, therefore, a crucial credential in food packaging applications. Pure PVA exhibits low tensile strength and high elongation at break, this is supported by the fact that the polymeric chains in pure PVA can show sliding movements easily by the application of external force. With the inculcation of osthol into PVA polymer, hydrogen-bonding interactions between the polymer and osthol functional groups could have restricted the movement of polymer chains by making the polymer chains tight and rigid. Tensile strength, therefore, shows an increment, and elongation at the break on the flip side shows a decreasing trend [31]. With the addition of boric acid as a cross-linking agent to PVA, an amelioration in the mechanical property was noted [69].

### 3.11. Biological studies

#### 3.11.1. Antibacterial activity

**Figure 10:**
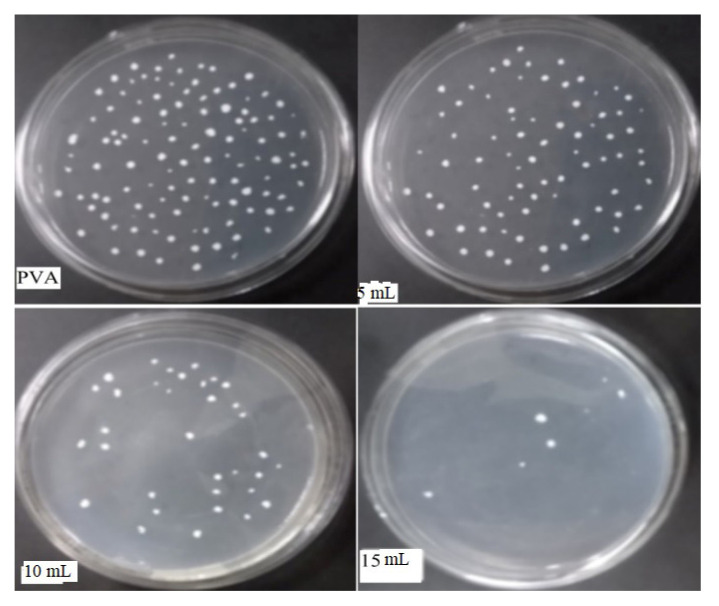
Antibacterial activities of films against *E. coli*.

**Figure 11:**
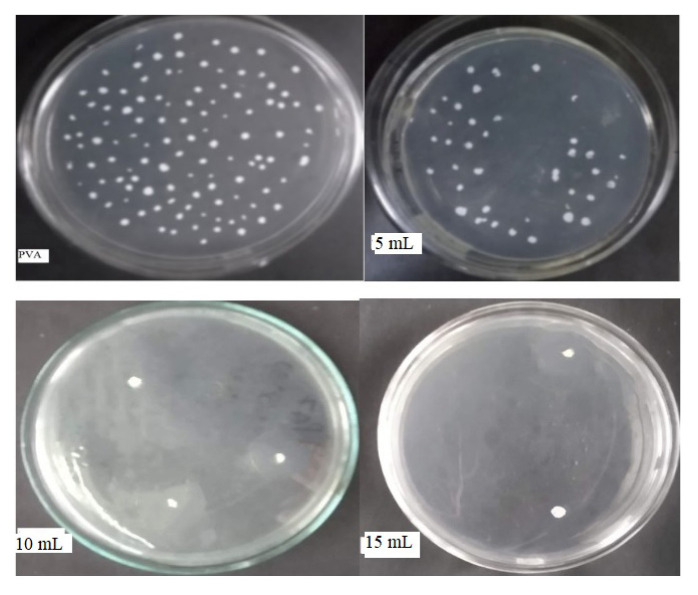
Antibacterial activities of films against *S. aureus*.

The antibacterial properties of polymer composite films against *S. aureus* and *E. coli* were evaluated by the liquid test culture method. The 15-mL polymer composite film inhibited the growth of both bacteria. The film composites containing 5 mL of osthol showed negligible antibacterial activity. The results obtained from the method are shown in Figures 10 and 11 against *E. coli* and *S. aureus*, respectively.

**Figure 12:**
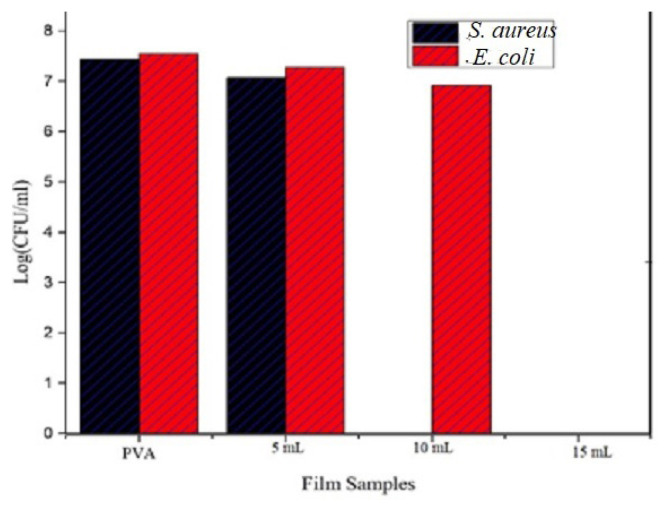
Log CFU/mL of composite films against *S. aureus* and *E. coli*.

Antimicrobial activity is aimed to minimize the growth of microbes in the food package. Antibacterial activity was confirmed by the colony count method by counting the number of viable cells, i.e. colonial forming units per millilitre. From the graph, as shown in Figure 12, which shows log CFU/mL against all-composite film samples, it is clear that 15-mL composite film has shown antimicrobial activity against both tested pathogens and 10-mL composite film has shown antimicrobial activity against *S. aureus*. This significant activity exhibited by the 15-mL film sample against both establishes the fact that the polymer composite film has good antibacterial properties and can be used for antimicrobial applications; hence, it could show its provision in active packaging antimicrobial films.

#### 3.11.2. Antifungal activity

**Figure 13:**
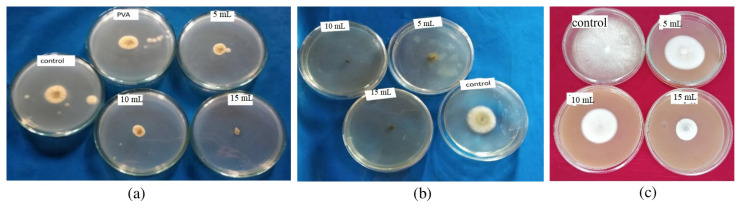
Antifungal activities of polymer composites of osthol against **(a)** *Asperigillus niger*, **(b)** *Alternaria alternate*, **(c)** *Fusarium solani*.

Composite films of osthol were evaluated for their efficacy on mycelial growth of pathogenic fungi *Aspergillus niger*, *Alternaria alternata*, and *Fusarium solani*by food poisoning technique. As indicated in Figure 13a and Table 3, we can see that the radial growth of *Aspergillus niger* is inhibited by increasing the concentration of osthol in composite films. In control, the mean diameter of mycelial growth is 24 ± 0.25 mm and in 15-mL composite diameter of mycelial growth is diminished to about 9.07 ± 0.15. Table 3 shows the same results where the diameter of mycelial growth is compared with the concentration (**μg/mL) and percentage inhibition** of osthol in the polymer composite. Similarly in Figures 13b and 13c and Tables 4 and 5, radial growth of *Alternaria alternate* and *Fusarium solani* was inhibited by increasing the concentration of osthol.

It is imperative from the tables that with increasing concentration of the osthol in the composite films, the mycelial growth is decreased. This inhibition of the mycelial growth by the composite films containing different amounts of osthol indicates that they show antifungal activity and hence can find application in the food packaging industry.

## 4. Conclusion

Biopolymers are commonly used to minimize environmental degradation. PVA is one of the most common synthetic biopolymer and is considered safe and environmentally friendly. In this research manuscript, a natural coumarin osthol was incorporated into the polymer PVA to improve its barrier and mechanical and antimicrobial properties for antimicrobial applications. The resultant PVA osthol composite films showed more water resistance due to an increase in hydrophobic character. This is reflected with the increase in contact angle of 15-mL composite film by 21.36° as compared to virgin PVA. The enhancement in the mechanical, thermal, antimicrobial, and barrier properties are appreciable in the films with the higher concentration of osthol (15 mL). Keeping in view the above encouraging results, such PVA osthol films can have wider applications in the food industry and can act as a potential candidate for food packaging applications.

## Figures and Tables

**Table 1:** Thickness, moisture content (MC), transparency values, and film solubility are given as mean ± standard deviation.

Samples	Thickness (mm)	Moisture content (MC)	Transparency	Film solubility (FS)
Pure PVA	0.126 ± 0.0020	11.23%	0.397 ± 0.006	99.11%
5-mL PO	0.143 ± 0.0015	8.86%	0.755 ± 0.008	93.21%
10-mL PO	0.153 ± 0.0025	7.02%	1.104 ± 0.018	63.19%
15-mL PO	0.156 ± 0.0015	6.67%	1.122 ± 0.011	57.42%

**Table 2:** OTR, WVTR, TS, and % EB of all Film samples.

Film samples	OTR (cc/m^2^/day)	WVTR (g/m^2^/day)	Tensile strength TS (MPa)	% elongation at break (EB)
PVA	9.04 ± 0.065	958.66 ± 3.858	18.73 ± 0.56	165± 2.45
5-mL	7.78 ± 0.041	808.33 ± 3.399	19.84 ± 0.72	147 ± 3.26
10-mL	6.11 ± 0.066	724.67 ± 3.091	22.51 ± 0.50	132 ± 2.05
15-mL	5.15 ± 0.041	668.67 ± 3.858	24.58 ± 0.49	123 ± 2.86

**Table 3:** Concentration of osthol in polymer composites vs mean diameter and % inhibition.

Mean diameter(mm)	Conc. (μg/ml)	% inhibition
24.07 ± 0.25	0	0
23.03 ± 0.30	0	4.32 %
16.13 ± 0.25	83	32.98%
14.03 ± 0.35	143	41.71%
9.07 ± 0.15	187	62.32%

**Table 4:** Concentration of osthol in polymer composites vs mean diameter and % inhibition.

Mean diameter (mm)	Conc. (μg/mL)	% inhibition
85.40 ± 0.40	0	0
50.03 ± 0.21	83	41.42
42.10 ± 0.26	143	50.70
30.10 ± 0.25	187	64.75

**Table 5:** Concentration of osthol in composite films vs the mean diameter and % inhibition.

Mean diameter (mm)	Conc. (μg/mL)	% inhibition
28.07 ± 0.15	0	0%
17.93 ± 0.25	82	36.12%
7.17 ± 0.21	142	74.46%
6.07 ± 0.25	186	78.37%

## Data Availability

The data generated during the current study are available with the corresponding author.
